# Changes in Microbial Community Structures under Reclaimed Water Replenishment Conditions

**DOI:** 10.3390/ijerph17041174

**Published:** 2020-02-12

**Authors:** Jie Li, Yujiao Sun, Xiaoyu Wang, Shangwei Xu

**Affiliations:** College of Water Sciences, Beijing Normal University, Beijing 100875, China

**Keywords:** reclaimed water, biofilm, replenishment, eutrophication, microbial community

## Abstract

Using reclaimed water as a resource for landscape water replenishment may alleviate the major problems of water resource shortages and water environment pollution. However, the safety of the reclaimed water and the risk of eutrophication caused by the reclaimed water replenishment are unclear to the public and to the research community. This study aimed to reveal the differences between natural water and reclaimed water and to discuss the rationality of reclaimed water replenishment from the perspective of microorganisms. The microbial community structures in natural water, reclaimed water and natural biofilms were analyzed, and the community succession was clarified along the ecological niches, water resources, fluidity and time using 16S rRNA gene amplicon sequencing. Primary biofilms without the original community were added to study the formation of microbial community structures under reclaimed water acclimation. The results showed that the difference caused by ecological niches was more than those caused by the fluidity of water and different water resources. No significant difference caused by the addition of reclaimed water was found in the microbial diversity and community structure. Based on the results of microbial analysis, reclaimed water replenishment is a feasible solution that can be used for supplying river water.

## 1. Introduction

In recent years, water resource shortages and water environmental pollution have become major problems all over the world, and China is no exception [1,2]. The pressure on fresh water supplies, not only for drinking but also for urban landscapes, is increasing especially for large and medium-sized cities [3]. It is urgent to take measures to ensure that the supply of water resources can meet the demands of urban development and growing populations [4,5]. Reclaimed water has become the first choice for replenishing urban landscape rivers. Successfully reclaimed water acquired after physical, chemical and biological treatments of domestic wastewater or municipal sewage must satisfy the national discharge standards [6]. However, successfully reclaimed water still contains nutrients, potentially hazardous compounds such as emerging contaminants, heavy metals and pathogens [7,8]. Therefore, the safety of water and the risk of eutrophication must be considered when utilizing reclaimed water.

As more reclaimed water is released into rivers, eutrophication caused by blue-green algae has become one of the largest concerns for city managers. Feng et al. believe that high concentrations of nitrogen and phosphorus contribute to the eutrophication of enclosed landscape water supplemented by reclaimed water [9]. Ao et al. studied three ponds replenished with reclaimed water and found a strong impact of reclaimed water to eutrophication [10]. The occurrence of eutrophication has the potential to cause the death of a large number of aquatic organisms, destroy aquatic functions and eventually affect the urban river landscape [11]. As an important part of aquatic ecosystems, aquatic microorganisms can directly reflect water quality and play an important role in the monitoring and early warning of water eutrophication [12]. More specifically, microbial community structure and succession are tightly associated with environmental factors [13,14] and may be used to characterize the physical and chemical features of water [12].

In addition to the microorganisms in water, biofilms also play a significant role in microbial-mediated biogeochemical processes in aquatic ecosystems [15]. Biofilms adsorb contaminants from the water impacted by organic and inorganic contaminants [16] and collect the inorganic particles, microbes and algae, which contribute to the base of the food web in rivers [17,18]. There is a high diversity and abundance of microbes living in biofilms [19]. Adequate nutrients from reclaimed water can guarantee the growth of biofilms and offer the microbial community functional potential [20]. Stable biofilms may effectively reflect the evolution of microbial community structures and functions associated with the aquatic environment [21].

In this study, it was explored whether reclaimed water supply for urban landscape rivers was feasible or would affect the ecological environment and increase the risk of eutrophication. In situ experiments have some limitations, including the release of pollutants and human disturbances. Therefore, an experiment without human interference was designed in the laboratory to observe the ecological change under reclaimed water replenishment conditions and to compare it with that of natural river water. The regeneration of microbes grown in different types of water was observed to further understand the microecological succession affected by the reclaimed water. This study provides theoretical support for the monitoring and management of reclaimed river water supply and water quality in the future.

## 2. Materials and Methods

### 2.1. Water Resources for the Experiment

Beijing, the capital of China, which has obvious regional characteristics and representativeness, was chosen as the experimental site and natural water sampling point for the reason that the reclaimed water supply channel was mainly located in large and medium-sized cities. Natural water (NW) was collected from an urban river named the Yongding River (116°4′27.52″ E 40°0′9.39″ N) with slight eutrophication, and reclaimed water (RW) was taken from the final effluent of a reclaimed water treatment plant in Beijing with an improved inverted A^2^/O treatment process. The experiment was carried out in the Experimental Base of Beijing Normal University (116°3′15.57″ E 39°41′27.79″ N). The experiment was carried out in summer. The temperature in this season met the requirements for the growth of most microbes, which was more conducive for the observation of microbial diversity [22]. A reasonable ≤ 80% reclaimed water replenishment ratio was proposed based on preliminary pilot experiments. Therefore, in this experiment, considering the introduction of algal species under natural conditions, and to make the experiment more consistent with the reality of urban river water replenishment, the proportion of 80% reclaimed water and 20% natural water was employed rather than 100% reclaimed water.

### 2.2. Experimental Design

**Figure 1:**
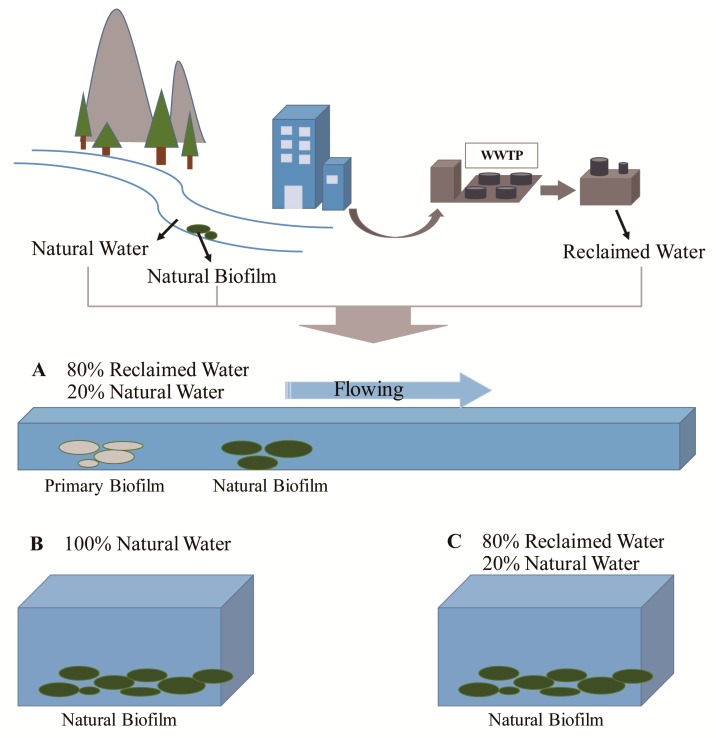
Water and biofilm collection and experimental design. (**A**) 80% reclaimed water, 20% natural water; (**B**) 100% natural water; (**C**) 80% reclaimed water, 20% natural water.

The original objective of the experiment was to compare the differences between reclaimed water and natural water. Considering that the fluidity of water (the water was flowing or still) will also affect the result and that the flowing water may exert a different effect on the microbes, another water tank (Figure 1A) was designed to make the experimental conditions close to the real river conditions. Ultimately, there were three devices in the entire experimental process (Figure 1). Device A (600 × 120 × 45 cm) was a flowing tank in which the water was a mix of 80% reclaimed water from the reclaimed water plant and 20% natural water from the river (there was no reclaimed water discharged into the river). The water in device A was recirculated, and no fresh water was pumped during the experimental period. Device B (45 × 31 × 27 cm) was a motionless water tank filled with 100% natural water collected from the river. Device C (45 × 31 × 27 cm) was also a motionless tank, but with 80% reclaimed water and 20% natural water.

To observe not only the change in water but also the change in biofilms, stones were collected from the river and placed into the bottom of each device for collecting biofilm, which was called natural biofilm (NF). To observe the complete primary biofilm formation process, sterilized clean stones were placed in device A with flowing water. The stones did not contain any microbial communities on the first day, and the biofilms that grew were completely dependent on the substances in device A. Thus, the biofilm of these stones was called primary biofilm (PF).

The experiment was carried out under normal sunlight, and the water level in the device decreased by 0.49 cm on average every day. The entire experimental period continued for 28 days. On the first day, the original communities of reclaimed water (RW), natural water (NW) and natural biofilm (NF) were detected. Later, water samples and biofilm samples were collected three times on days 10, 19 and 28. Devices and sample names are explained in Figure 1 and Table S1.

### 2.3. Sample Collection and Preprocessing

Water samples were collected into one liter sterile bottles. Biofilm samples were collected from the stone surface with the same unit area and collected into sterilized 2 mL centrifuge tubes. A sterilized ring with a diameter of 3.4 cm was used to cover the surface of stones, and the biofilm in the ring was collected using a sterilized knife to guarantee the same unit area biofilm samples. Manipulation instruments, glassware and tubes were autoclaved prior to use, and the surface of instruments was cleaned by wiping with 75% ethanol before each sampling. All samples were taken in triplicate and quickly transported to the laboratory. Water samples that were used for the determination of chemical properties were kept in a 4 °C refrigerator and were detected within 24 h, and other water samples were filtered through a 0.22 μm mixed cellulose ester water filter (Jinjing Brand, Shanghai, China) within 24 h of collection [23]. The membranes and biofilms were stored in a freezer for preservation at −20 °C until subsequent DNA extraction [24].

### 2.4. Water Quality and Analytical Methods

The pH was determined using a handheld pH meter (PHscan 10S, Shanghai, China). A YSI-556 multiparameter water quality tester (YSI, Ohio, OH, USA) was used to detect the water temperature (T), electric conductivity and dissolved oxygen (DO). Chemical oxygen demand (COD) was determined by rapid digestion spectrophotometry (HJ/T 399-2007, China), and ammonia nitrogen was detected according to Nessler’s reagent spectrophotometry (HJ 535-2009, China). Water samples were filtered through 0.45 μm mixed fiber Millipore filters (Jinjing Brand, Shanghai, China, diameter 50 mm) for the detection of NO_3_^−^ (nitrate), PO_4_^3−^ (phosphate), Cl^−^, SO_4_^2−^, Na^+^, K^+^, Mg^2+^ and Ca^2+^. Cationic chromatography (Dionex Aquion, Thermo Scientific, Massachusetts, USA) and anion chromatography (Dionex ICS-2100, Thermo Scientific, Massachusetts, MA, USA) were used separately for the detection of the abovementioned parameters.

### 2.5. Genomic DNA Extraction and High-Throughput Sequencing

Before DNA extraction, the filtered membranes were cut and placed into a centrifuge tube and then followed by the extraction method using cetyltrimethyl ammonium bromide (CTAB) [25]. The whole process of DNA extraction was performed on a bacteria-free workbench with UV irradiation before operation. DNA quality and concentration were measured by gel electrophoresis and a Nanodrop spectrophotometer (Nanodrop 2000, Thermo Scientific, Wilmington, USA), respectively [26]. Extracted DNA was stored at −20 °C and then sent to Shanghai Majorbio Bio-pharm Technology Company (China) for sequencing. The V3–V4 regions of the 16S rRNA gene were amplified using the primer pair 338F (5’-ACTCCTACGGGAGGCAGCAG-3’) and 806R (5’-GGACTACHVGGGTWTCTAAT-3’), which target conserved sequences found in bacteria [27,28].

### 2.6. Statistical Analysis

Pair-end sequence data were acquired with the Illumina MiSeq platform (PE 300, Illumina, San Diego, USA), and raw data were quality filtered using Trimmomatic [29,30]. Low-quality reads with scores <20 were discarded with a sliding window of 50 bp [31]. Then, barcodes were matched, and unmatched reads were removed. Samples were distinguished according to the barcodes and primers at both ends of the sequence, and the sequence direction was adjusted to finally obtain the optimized sequences. The taxonomy of sequences was analyzed by RDP Classifier (http://rdp.cme.msu.edu/) [32,33] according to the SILVA (Release 128) rRNA database (http://www.arb-silva.de).

Alpha diversity including species richness (OTUs, operational taxonomic units) and Chao index, which were used to calculate the community richness, and Shannon index and Simpson index, which represented the community diversity [34], were analyzed through Mothur (V 1.30) [35]. Student’s *t*-test was used to test the Shannon index of the microbial communities from different groups by R software. The difference in microbial taxa in groups could be explained by the Venn diagram at the OTU level at 97% identity, representing the unique OTU of one group and the intersection of two or more OTUs [36]. Principal coordinates analysis (PCoA) was used to visualize the community structure among groups [37]. The PCoA plot was generated from the Bray–Curtis similarity index. Analysis of similarities (ANOSIM) was used to test differences in bacterial community composition among groups [38]. These analyses were run in R software with the vegan and ggplot2 packages.

The correlations between the microbial community and environmental factors were observed based on the redundancy analysis (RDA) with the help of Canoco software for Windows 4.5. There were many environmental factors related to the composition of the sample species, but many of the factors were correlated with one another. Thus, environmental factors were selected by the functions of the VIF (variance inflation factor) [30] after judging the collinearity among different factors [39], and DO and Mg^2+^ were removed in the following analysis for VIFs higher than 10.

## 3. Results and Discussion

### 3.1. Water Quality

The water quality parameters of the different devices are shown in Table 1. The temperature fluctuated between 27.2 and 29.1 °C. No significant change was found when comparing the pH, COD and DO. However, it was obvious that the concentrations of nutrients such as ammonia nitrogen, nitrate and phosphate were lower in device B (100% natural water) than in device A and C (80% reclaimed water). It confirmed that the nutrient content in reclaimed water was high, and this indicated that a high nutrient concentration was an important indicator of reclaimed water replenishment different from that of natural water replenishment. The concentration of nutrients in the reclaimed water device was comparable to that in polluted urban rivers [40]. Nutrient pollution caused by excess loadings of nitrogen and phosphorous has been widely observed [41] and may lead to the changes of water physicochemical parameters and aquatic biodiversity [42]. Additionally, the concentrations of K^+^, Cl^−^ and Ca^2+^ were lower in device B than in devices A and C.

### 3.2. Sequencing Assessment and Taxonomic Diversity

In total, 1,673,754 optimized sequences were generated from 25 samples. The length distribution of trimmed sequences ranged from 421 to 460 bp, with an average of 436 bp. According to the minimum sequence number of a certain copy, the sequence number of all samples was randomly selected to the same level, and the homogenization data were used for subsequent analysis [43]. After subsampling to an equal sequencing depth (22,789 reads per sample) and clustering, 4785 OTUs (operational taxonomic units) at 97% identity were obtained. A rarefaction curve was used to indicate the coverage of the sequencing and enabled the assessment of differences in species richness among different samples (Figure S1). All curves in the figure flattened after the sequences reached a relatively large number.

Shannon, Simpson and Chao indices representing the alpha diversity were calculated at the OTU level (Table 2) [12,34]. The Shannon index of biofilm was higher than that of water, and the Simpson index of biofilm was lower than that of water, indicating that the alpha diversity of biofilm was higher than that of water. The community richness calculated by the Chao index was also higher in biofilm. The lowest Shannon index value of biofilm was found on day 28 of device B (natural biofilm), and the Shannon index was also found to be low in water at the same time.

**Figure 2:**
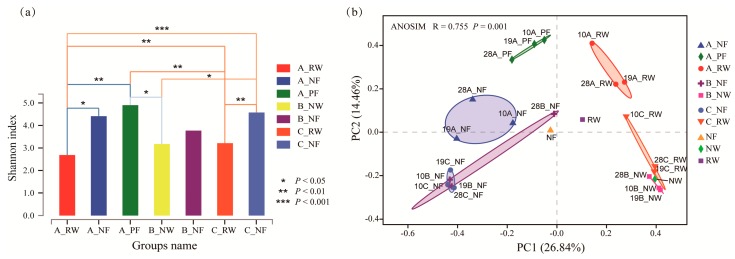
Alpha and beta diversity of the microbial communities from different groups. (**a**) Shannon index of alpha diversity and differences among groups (*: *p* < 0.05, **: *p* <0.01 and ***: *p* < 0.001); (**b**) principal coordinates analysis (PCoA) of the bacteria community in different group samplings based on ecological niches and devices. ANOSIM was used to test the significance of variations.

The alpha diversity was investigated in different groups divided according to the different devices (A, B and C) and ecological niches (water and biofilm) (Figure 2a). Higher biodiversity is assumed to have a stronger tolerance to external environmental pressures, while the decrease of diversity would impair the function of ecosystems [44,45]. Unexpectedly, there was no significant variation in the diversity between devices B and C with different water resources (*p* = 0.9246). This may indicate that the addition of 80% reclaimed water did not significantly affect microbial diversity. However, by comparing the devices A and C, both with reclaimed water, it was found that there was no significant difference between biofilms (A_NF and C_NF) (*p* = 0.7779), but a significant difference was found between water (A_RW and C_RW) (*p* = 0.0012), indicating that the fluidity of water changed the diversity in the water rather than the biofilm. This may demonstrate that the stable microbial community structure of natural biofilms was hardly influenced by changes in the external environment, such as water resources and fluidity conditions. It was obvious that the diversity in reclaimed water in the flowing tank A(A_RW) was significantly lower than those in natural biofilms (A_NF) (*p* = 0.0280) and primary biofilms (A_PF) (*p* = 0.0019). Similarly, the diversity in reclaimed water in still tank C (C_RW) was also significantly lower than those in biofilms (C_NF) (*p* = 0.0018). Although the diversity in water in device B (B_NW) was also lower than those in the biofilms (B_NF), the difference was not significant (*p* = 0.5410). This may be attributed to the fact that the natural water and biofilm in B were collected at the same site, and a stable microecological system was established depending on the interaction between water and biofilm over a long time.

The beta diversity was also calculated for the different groups (Figure 2b). The results of the PCoA (principal coordinates analysis) showed the community distribution of the samples [46,47]. The main coordinates explained 26.84% and 14.46% of the total variation in bacterial data. The ANOSIM was used to compare the mean of ranked dissimilarities between groups to the dissimilarities within groups. A high R value (R = 0.755) close to “1” indicated a strong compositional difference between groups (*p* = 0.001). It could be seen from the plot that there were obvious differences between different ecological niches, and all samples were divided into two clear groups: water groups and biofilm groups. Although there were still differences between flowing water and still water, the differences were smaller than those caused by different ecological niches. At the same time, it was observed that although device B contained completely natural water and device C contained mostly reclaimed water (80%), their microbial community structures were very similar. Therefore, it can be considered that microorganisms cultured in reclaimed water are not very different from those cultured in natural water. From the perspective of microorganisms studied in this experiment, replacing part of the natural water in landscape rivers with reclaimed water may be a feasible solution for water resource shortage and exhausted rivers.

### 3.3. Common and Unique Microbial Taxa of Different Groups

**Figure 3:**
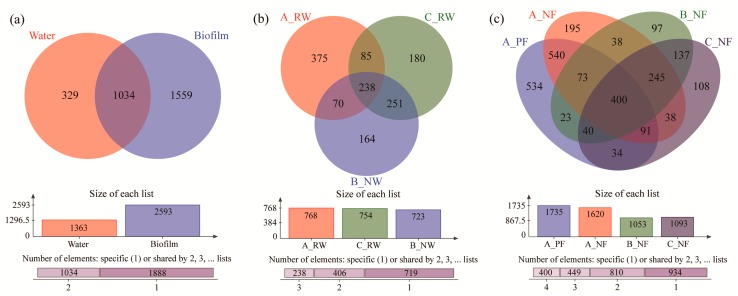
The microbial taxa of different groups at the OTU level. (**a**) The differences between water and biofilm; (**b**) water in different devices; (**c**) biofilms in different devices.

There were 1034 OTUs shared between the water and biofilm (Figure 3a), indicating that they might be insensitive to different ecological niches. The unique OTUs in biofilm were significantly more than those in water, which explained the higher diversity of the biofilm. For the water (Figure 3b) in different devices, flowing reclaimed water held the largest number of unique OTUs (375), and the number of total OTUs in the three different devices was similar. Devices B and C had more shared OTUs than other samples despite the different water sources, and this result demonstrated that the community was similar in still natural and reclaimed water. Therefore, it was concluded that the microbial community difference caused by fluidity was more than that caused by different water resources. For biofilms (Figure 3c) in different devices, primary biofilm had the highest numbers of total OTUs (1735) and unique OTUs (534). The number of unique OTUs in A_NF (195) was also more than those in B_NF (97) and C_NF (108), indicating that the fluidity also had a noticeable influence in not only water but also biofilms.

### 3.4. Bacterial Community Succession in Different Ecological Niches

#### 3.4.1. Water

**Figure 4:**
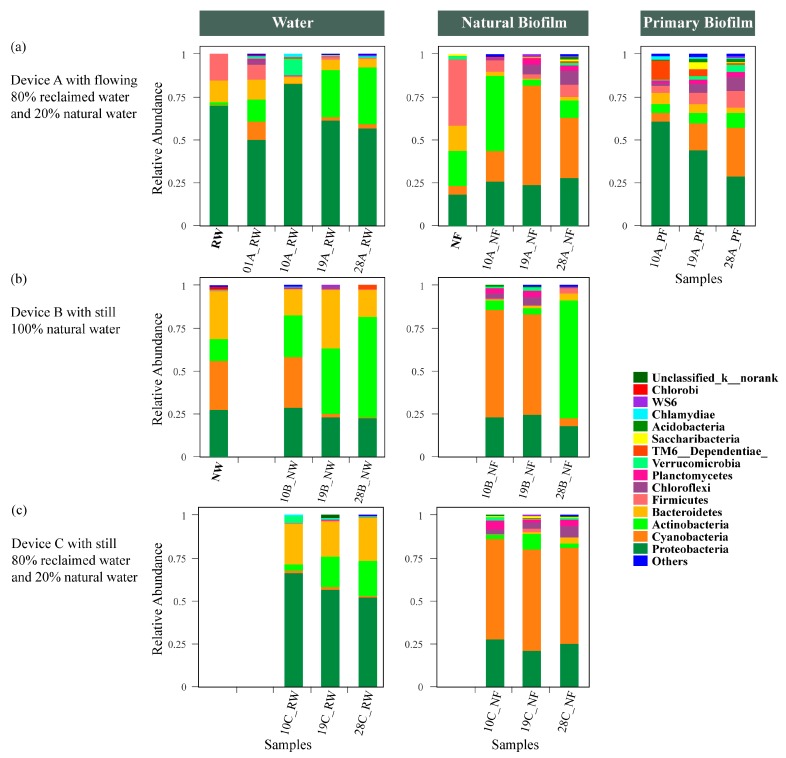
Composition of bacterial community structure. (**a**) Device A with flowing 80% reclaimed water and 20% natural water; (**b**) device B with still 100% natural water; (**c**) device C with still 80% reclaimed water and 20% natural water.

For the source of water used in this study, the phyla with the highest relative abundance in reclaimed water (RW) were Proteobacteria (69.71%), Firmicutes (14.99%) and Bacteroidetes (13.01%) (Figure 4a). The main characteristic of reclaimed water was that it contained the highest abundance of Proteobacteria. Proteobacteria have been reported as the most widespread bacteria in the active sludge community of sewage treatment plants [48], and many species of this phylum pose a potential risk to human health [26]. The predominant phyla (generally, in this study, predominant phyla had a relative abundance >10.00%) of natural water (NW) were Cyanobacteria (29.01%), Bacteroidetes (27.84%), Proteobacteria (27.36%) and Actinobacteria (12.63%) (Figure 4b). Cyanobacteria have a long-recognized ecological importance in freshwater [49]. In a study on the change in the bacterial community in the eutrophication area of Dong Lake in different seasons, Cyanobacteria became the most dominant phylum in August [50], which is similar to this result.

In device A, the predominant phyla were Proteobacteria, Actinobacteria and Bacteroidetes. On day 10 (10A_RW), the relative abundance of Proteobacteria increased suddenly, but Actinobacteria and Cyanobacteria decreased to the lowest abundance. Proteobacteria have been confirmed to be typical and dominant freshwater microbes in aquatic habitats, including rivers and lakes [51]. Proteobacteria may adapt well to changes in the environment when fluidity increases compared to the habitat they lived in before [52]. Interestingly, on this day, Verrucomicrobia also increased, and the abundance (9.49%) ranked only second to Proteobacteria with the highest abundance. Verrucomicrobia are widespread in lakes and rivers, but their roles are not well understood [53].

The predominant phyla of device B were Proteobacteria, Actinobacteria, Cyanobacteria and Bacteroidetes. Cyanobacteria had almost no change between day 1 and day 10 (NW and 10B_NW). The water was still in the state of eutrophication. However, the abundance of Cyanobacteria rapidly decreased on day 19 (19B_NW), and Cyanobacteria were no longer the dominant microbes. When Cyanobacteria decreased to the lowest value, Actinobacteria became the predominant phylum in water and even had a high proportion of 58.76% on 28B_NW. In the whole changing process of device B with 100% natural water, the relative abundance of Proteobacteria remained stable. Although the original water was eutrophic, the abundance of Cyanobacteria decreased and no longer dominated the eutrophic water environment because of the increase in Actinobacteria, without the import of exogenous sources [54].

The abundance of Actinobacteria from water in device C (Figure 4c) increased from day 10 to day 28, while that of Proteobacteria gradually decreased. In addition to the two dominant phyla, the number of Bacteroidetes, another dominant phylum, basically maintained a constant state. This variation trend was similar to device A (flowing reclaimed water), although the difference in diversity was significant (Figure 2a). In contrast to the highly abundant Cyanobacteria obtained from the first two samples in device B, the number of Cyanobacteria in device C was stable and low in abundance, indicating a lower risk of eutrophication [55].

#### 3.4.2. Natural Biofilm and Primary Biofilm

The dominant microbes of natural biofilms collected in the river channel on the first day (NF) were Firmicutes (38.61%), Actinobacteria (20.69%), Proteobacteria (18.27%), Bacteroidetes (14.74%) and Cyanobacteria (4.95%) (Figure 4a). There are few reports about Firmicutes as the most dominant phylum in biofilms, and a high relative abundance of Firmicutes is usually detected in wastewater [56]. This may indicate that the water or biofilm may be contaminated. However, after a period of incubation in device A, Firmicutes was no longer the predominant phylum in biofilms and was replaced by Actinobacteria (43.81%) (10A_NF), which was then replaced by Cyanobacteria (19A_NF and 28A_NF). For devices B and C, the changes in the biofilm community structure were similar, despite the water being different. On day 10, Cyanobacteria became the most abundant phylum in the natural biofilm, changing the dominant positions of Firmicutes, Actinobacteria, Proteobacteria and Bacteroidetes. Planctomycetes and Chloroflexi were also present and occupied a certain proportion. Inexplicably, the Actinobacteria in device B increased dramatically on day 28 (28B_NF). At the same time, Actinobacteria also presented a high abundance in water (28B_NW). However, unlike the rapid increase in biofilm, the change in Actinobacteria in natural water was gradual. The mechanism of interaction between Actinobacteria and Cyanobacteria remains unclear. Studies have shown that Actinobacteria can lyse Cyanobacteria by releasing extracellular substances such as l-lysine, and others have found that Actinobacteria are more abundant in water with lower eutrophication [57,58,59]. It may be inferred that the rapid increase in biofilm was influenced by water; filamentous Actinobacteria [60] proliferate in the water first, and when they reach a certain abundance, they deposit on the surface of the biofilm at the bottom, causing a sharp decrease in the number of Cyanobacteria because of limited photosynthesis or the pressure of competition. This can also be used to explain why the lowest Shannon index value of biofilm was found on day 28 (Table 2). 

The alpha diversity of the primary biofilm was higher than that of the natural biofilm in device A for each day. On day 10, Proteobacteria (60.74%) and TM6__Dependentiae (11.29%) were the predominant phyla, while the numbers of these two phyla decreased gradually over time. Subsequently, Cyanobacteria, Actinobacteria, Firmicutes and Chloroflexi increased and became more abundant phyla. At the same time, the diversity measured by the Shannon index was also increased (Table 2). Proteobacteria were widespread in the water and soil environment. It was inferred that the generation of microorganisms on the primary biofilm was influenced by the community in water. Easy-growing microorganisms such as Proteobacteria settled first, creating proper conditions for others, and built stable community structures with the change in the environment. For natural biofilms, microbes already established stable systems to resist changes in the external environment [19]. Thus, compared with natural biofilms, the microbial community structure of the primary biofilms changed more regularly over time.

In summary, the relative abundance of Cyanobacteria in water was lower than that in biofilms, either still or flowing, natural or reclaimed water (Figure 4). While the number of Cyanobacteria in the water decreased, the number of Cyanobacteria in the biofilm did not decrease significantly. Even in the primary biofilms without the original community, the relative abundance of Cyanobacteria was increasing. This may be due to the large amount of Cyanobacteria deposited at the bottom in the early stage of bloom, and only when the proper external conditions are available will they rise to the surface and erupt in large numbers, eventually leading to eutrophication [61]. Therefore, the monitoring and early warning of water eutrophication should not be limited to the monitoring of water, and the monitoring of biofilms and sediments closely related to water will play a vital role in the early warning of water blooms. 

### 3.5. Correlation Between Microbial Community and Environmental Factors

**Figure 5:**
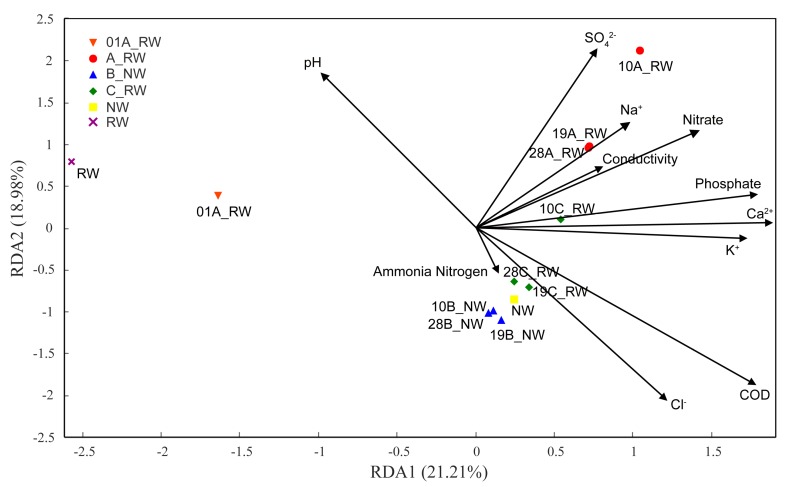
Correlation between microbial community and environmental factors.

The relationship between the microbial community and different environmental factors was always positively correlated (Figure 5). Nutrients including ammonia nitrogen, nitrate and phosphate had a great influence in different samples. In addition, nitrate had a closely positive correlation with flowing reclaimed water in device A, and ammonia nitrogen had a closely positive correlation with still water in devices B and C. For inorganic ions, Na^+^ and SO_4_^2−^ were closely correlated with flowing reclaimed water; however, Cl^-^ was correlated with the still water in devices B and C. Nitrogen nutrients have been suggested to be the dominant factors affecting the community structure in the Dongjiang River [51] and the tributary of the Three Gorges reservoir [62]. Nitrogen fixation, ammoniation, nitrification and denitrification in nature are all inseparable from the participation of microorganisms [63]. Other studies found that phosphate was the main environmental factor influencing the structure of the bacterial community in surface water, such as reservoirs [56] and rivers [64], because bacteria can assimilate phosphate through the cell membranes to meet their need for phosphorus [65]. No specific relationship caused by the environmental factors was found between natural water and reclaimed water in this study.

## 4. Conclusions

In this study, the microbial community structures in natural water, reclaimed water and natural biofilm were revealed, and the community succession was clarified along the ecological niches, water resources, fluidity and time. It was found that the diversity in biofilm was higher than that in water and that the diversity in primary biofilm was higher than that in natural biofilm. The results showed that the difference caused by ecological niches was more than that caused by fluidity of water and different water resources. No significant difference caused by the addition of reclaimed water was found in the microbial diversity and community structure. It can be hypothesized that the combination of 80% reclaimed water and 20% natural water was a feasible solution that could be used for supplying river water. In this research, we innovatively introduced the study of biofilm and proposed that the monitoring and early warning of eutrophication should not be limited to the monitoring of water, and the monitoring of biofilms and sediments closely related to water will play a vital role in the early warning of water blooms. This research was only conducted in summer, and this was a limitation because the conditions may be different in winter, which may affect the overall conclusion. Long-term and large-scale experiments need to be completed to better understand the succession of microbial community structures in different ecological niche conditions and the influence of reclaimed water on eutrophication.

## Figures and Tables

**Table 1:** Water quality parameters in different devices (A, B and C).

		A	B	C
Temperature (°C)	max	29.1	28.0	27.9
	min	28.1	27.2	27.2
pH	max	9.4	9.9	9.8
	min	9.1	9.6	9.6
Chemical oxygen demand (COD; mg/L)	max	31	49	25
	min	20	20	15
Dissolved oxygen (DO; mg/L)	max	9.40	11.29	12.63
	min	6.78	8.92	9.42
Nitrate (mg/L)	max	190.60	2.06	51.98
	min	43.56	0.51	42.66
Phosphate (mg/L)	max	0.51	0	0.54
	min	0	0	0.27
Ammonia nitrogen (mg/L)	max	0.24	0.18	0.24
	min	0.13	0	0.02
Conductivity (S/m)	max	0.166	0.118	0.129
	min	0.091	0.091	0.091
Na^+^ (mg/L)	max	168.19	173.99	183.51
	min	98.67	116.90	128.98
K^+^ (mg/L)	max	19.53	10.96	21.62
	min	12.83	7.19	15.36
Mg^2+^ (mg/L)	max	49.35	41.77	33.97
	min	28.30	34.70	31.22
Ca^2+^ (mg/L)	max	118.11	27.90	46.22
	min	65.35	24.34	32.55
Cl^−^ (mg/L)	max	282.80	189.78	240.87
	min	131.82	123.07	163.90
SO_4_^2^^−^ (mg/L)	max	292.04	231.11	201.39
	min	119.46	160.95	144.67

**Table 2:** Diversity and richness estimated by Shannon, Simpson and Chao indices.

Sample	Operational Taxonomic Units (OTUs)	Shannon	Simpson	Chao
Natural water (NW)	373	3.45	0.07	691.82
Reclaimed water (RW)	279	2.52	0.14	453.15
01A_RW	731	4.24	0.04	926.71
10A_RW	324	2.68	0.17	511.64
19A_RW	473	2.62	0.16	705.60
28A_RW	419	2.76	0.14	687.83
10B_NW	492	3.78	0.05	819.54
19B_NW	372	3.19	0.08	649.22
28B_NW	337	2.56	0.25	603.22
10C_RW	442	3.16	0.08	679.78
19C_RW	422	3.31	0.07	714.97
28C_RW	411	3.17	0.08	660.35
Natural biofilm (NF)	405	3.74	0.05	508.26
10A_NF	530	3.48	0.16	591.33
19A_NF	835	4.53	0.04	1100.04
28A_NF	1236	5.23	0.02	1739.22
10A_PF	804	4.30	0.04	1205.37
19A_PF	1126	5.16	0.02	1483.47
28A_PF	1272	5.25	0.02	1595.97
10B_NF	673	4.60	0.02	898.18
19B_NF	737	4.57	0.04	952.36
28B_NF	204	2.14	0.44	206.63
10C_NF	545	4.26	0.03	718.04
19C_NF	751	4.88	0.02	846.21
28C_NF	684	4.59	0.04	815.68

## Supplementary Materials

The following are available online at https://www.mdpi.com/1660-4601/17/4/1174/s1, Table S1: Sample name and description, Figure S1: Rarefaction curve of water and biofilm samples.
