# Supplementary material for: Changes in Microbial Community Structures under Reclaimed Water Replenishment Conditions
Source: Int J Environ Res Public Health. 2020 Feb 12;17(4):1174. doi: 10.3390/ijerph17041174 (PMC7068412; doi:10.3390/ijerph17041174)
Supplement: Supplementary file 1 [file ijerph-17-01174-s001.pdf]

Supplementary Materials

Table S1. Sample name and description.

Sample	Description
NW	Natural water collected from the urban river
RW	Reclaimed water collected from the reclaimed water treatment plant
01A_RW	80% reclaimed water and 20% natural water from device A on day_01
10A_RW	80% reclaimed water and 20% natural water from device A on day_10
19A_RW	80% reclaimed water and 20% natural water from device A on day_19
28A_RW	80% reclaimed water and 20% natural water from device A on day_28
10B_NW	100% natural water from device B on day_10
19B_NW	100% natural water from device B on day_19
28B_NW	100% natural water from device B on day_28
10C_RW	80% reclaimed water and 20% natural water from device C on day_10
19C_RW	80% reclaimed water and 20% natural water from device C on day_19
28C_RW	80% reclaimed water and 20% natural water from device C on day_28
NF	Natural biofilms collected from the urban river
10A_NF	Natural biofilms from device A on day_10
19A_NF	Natural biofilms from device A on day_19
28A_NF	Natural biofilms from device A on day_28
10A_PF	Primary biofilms from device A on day_10
19A_PF	Primary biofilms from device A on day_19
28A_PF	Primary biofilms from device A on day_28
10B_NF	Natural biofilms from device B on day_10
19B_NF	Natural biofilms from device B on day_19
28B_NF	Natural biofilms from device B on day_28
10C_NF	Natural biofilms from device C on day_10
19C_NF	Natural biofilms from device C on day_19
28C_NF	Natural biofilms from device C on day_28

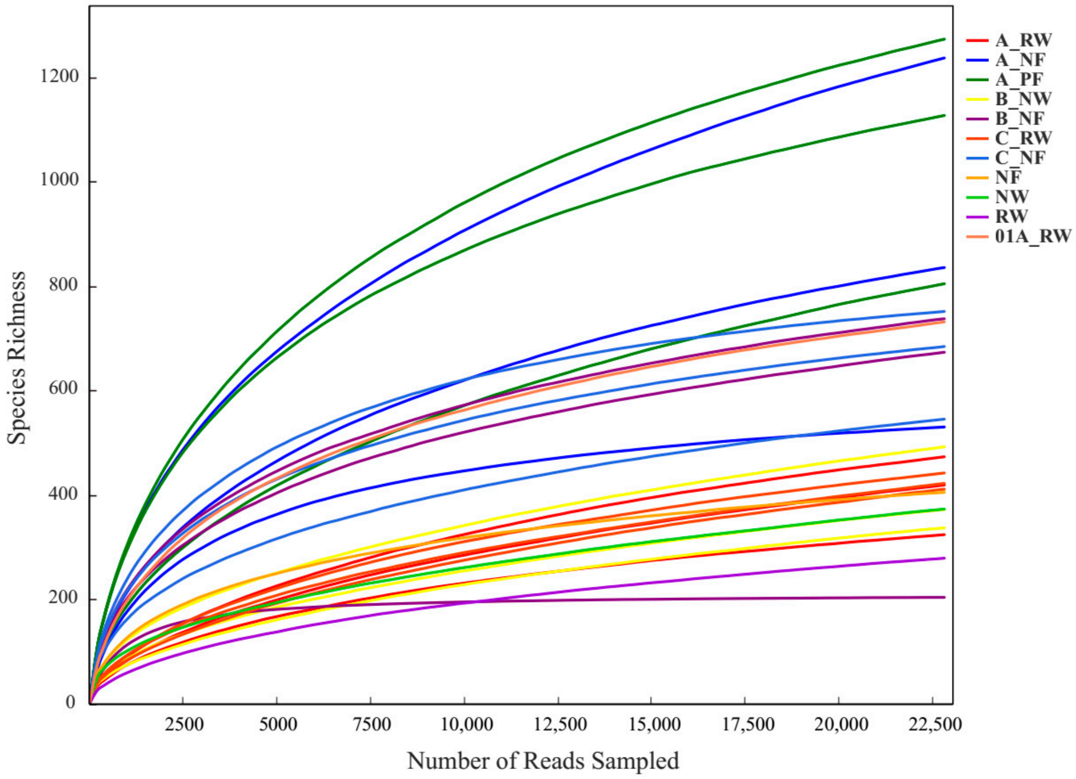

Figure S1. Rarefaction curve of water and biofilm samples.
